# Pattern of road traffic injuries in Yemen: a hospital-based study

**DOI:** 10.11604/pamj.2018.29.145.12974

**Published:** 2018-03-05

**Authors:** Eshrak Alfalahi, Ali Assabri, Yousef Khader

**Affiliations:** 1 Ministry of Public Health and Population Yemen Field Epidemiology Training Program Almaqaleh St, Sanaa city, Yemen; 2 Sana’a University, Faculty of Medicine and Health Sciences, Y-FETP Epidemiology Consultant, Department of Community Medicine and Public Health Flat No 63, New Stony Building, Sana’a University Compass, Sana’a, Yemen; 3 Jordan University of Science and Technology Department of Public Health Irbid, 22110, Jordan

**Keywords:** road traffic injuries, road traffic crashes, injuries, Yemen

## Abstract

**Introduction:**

Road traffic injuries (RTIs) are the eighth leading cause of death globally and the leading cause of death for young people. This study aimed to present time-limited trial surveillance in two referral hospitals to describe the pattern of RTIs in Sana'a, Yemen and determine road traffic crashes (RTCs) associated factors.

**Methods:**

All RTIs presented to Emergency Departments (ED) of the two Sanaa city hospitals between August and October, 2015 were studied and described. Data were collected everyday by trained data collectors. A pretested questionnaire modified from WHO injury surveillance form was used for data collection.

**Results:**

A total of 156 casualties from 128 RTCs had attended the two study hospitals during the study period. About 73% of victims were less than 30 years old. Only 13% of casualties were transported by ambulance. None of the victims wore the seat belt in case of 4-wheeled vehicles' users nor the helmet in case of 2-wheeled vehicles' users. Poor driving skills were involved in 133 (85%) casualties. Factors related to the vehicles contributed to 12% of RTCs. Of the 156 casualties, 17% had severe injuries and needed ICU admission. After 48 hours of the accident, 38% of patients ended with disability due to fractured limbs, 29% were not improving and their conditions were deteriorating, 18% had recovered and 5% died.

**Conclusion:**

Several personal, behavioral, environmental and vehicles related factors had contributed to RTIs in Yemen. The burden of RTIs in Yemen in terms of disability and mortality is high.

## Introduction

Road traffic injuries (RTIs) are the eighth leading cause of death globally and the leading cause of death for young people aged 15-29 [1]. Over million people worldwide died because of road traffic injuries [2]. More than 90% of all road traffic injuries' deaths occurred in the low and middle-income countries [3], with about one third was among pedestrians and cyclists. However, less than 35% of low-and middle-income countries have policies in place to protect road users [4]. Eighty-eight countries have reduced the number of deaths on their roads-but the total number of road traffic deaths remains unacceptably high at 1.24 million per year. There are large disparities in road traffic death rates between WHO regions. The Eastern Mediterranean region comes next to the African region with a death rate of 19.9/100,000 population compared to 26.6/100,000 in the African region [4]. Only 28 countries, representing 449 million people (7% of the world's population), have adequate laws addressing the most important related factors, namely speed, drink-driving, helmets, seat-belts and child restraints [4]. According to the global status report on road safety (2013) [2], the road traffic death rate in Yemen was 23.7 deaths per 100,000 population. Unfortunately, there were no data available for deaths by road-user category [4]. Furthermore, the source of these estimates was the Ministry of Interior (MOI) not the Ministry of Public Health and Population (MOPHP) as there is no well-established surveillance system in the MOPHP. Moreover, there are no established committees or bodies to address injuries issue in general and to prevent their occurrence and manage their consequences. This study aimed to present time-limited trial surveillance in two referral hospitals to describe the pattern of RTIs in Sana'a, Yemen. Moreover, the study aimed to determine road traffic crashes (RTCs) associated factors. The results of this study will be necessary for surveillance planning in MOPH.

## Methods

This study was conducted in Aljomhoury General Hospital and Science and Technology Private Hospital, between 24^th^ August 2015 and 8^th^ October 2015. All traffic injuries attended the two selected hospitals during the day and the night shifts of the study period were included unless overwhelmed by the number of the crashes coming at once; in such case, the first one at least was included. Data were collected everyday by the trained data collectors in the Emergency Departments (EDs) of the selected hospitals. The study questionnaire was developed based on the WHO injury surveillance form [5, 6] and was used for interviewing the study subjects. Where condition of victims did not permit the interview, the relatives of the victim were interviewed. During the interview, the purpose of study was explained to each respondent. Case-sheets of the victims were referred for cross-checking. The collected information consisted of personal identification data, history of road traffic crashes, human and environmental risk factors, clinical history and examination. The type and severity of injury suffered by the victims was graded using the injury severity scoring used by WHO guideline for injury surveillance [5]. The injury outcomes were also recorded for each case. For the purpose of study, RTC was defined as a collision or incident that may or may not lead to injury, occurring on a public road and involving at least one moving vehicle. RTI was defined as any injury due to crashes originating, terminating or involving a vehicle partially or fully in a public pathway [5]. A declaration about the researcher, the study objectives and its anticipated benefits was manifested at the beginning of each questionnaire. Oral informed consent was obtained from each conscious injured and, from the available accompanied relative, in case of unconsciousness or deceased. Along with that in case of child victims, asset was obtained. Confidentiality was protected for all participants. The study was approved by the ethical committee in the MOPHP. Data were described and analyzed using SPSS IBM version 20. Data were described using means for continuous variables and percentages for categorical variables.

## Results

**Characteristics of casualties**: A total of 156 casualties (82% males and 18% females) from 128 different road traffic accidents had attended the two study hospitals in Yemen during the study period. The distribution of causalities according to demographic and relevant characteristics is shown in Table 1. The average age of victims was 23 year. About three quarters of victims (73%) were less than 30 years old. About 39% of causalities were school pupils and pre-school children. About two thirds (62%) of casualties resulted from RTCs in Sanaa city, while the remaining 38% of casualties resulted from RTCs in other governorates. About 87% of the accidents occurred during the day time. The highest number of casualties caused by RTCs at 10 am (13%), at 12 noon (12%) and at 4 pm (10%). Fridays witnessed the highest numbers of casualties (19%) followed by Sundays (17%). The study revealed that 4% of the victims had a past history of traffic injuries and 4% reported chronic diseases history.

**Table 1:** The distribution of 156 casualties according to demographic and relevant characteristics

Variable	n	%
**Gender**		
Male	128	82.1
Female	28	17.9
**Age (year)**		
0 - <10	30	19.2
10 - <20	31	19.9
20 - <30	53	34.0
30 - <40	25	16.0
40 - <50	3	1.9
50 - <60	11	7.1
60 - <70	3	1.9
**Occupation**		
School pupils	37	23.7
Public employee	27	17.3
Pre-school age	24	15.4
Daily worker	20	12.8
Private employee	11	7.1
University students	10	6.4
Unemployed	10	6.4
Farmer	9	5.8
Business owner	8	5.1
**Category of road user**		
Drivers	47	30.1
Occupants	59	37.8
Pedestrians	50	32.1

**Distribution of injuries according the road user categories and the type of accident crash**: About 38% of injured people were occupants of vehicles other than drivers, 32% were pedestrians and the rest were the drivers. Of the drivers, 55% were motorized 2-wheeler drivers, 26% were 4-wheeler drivers and the rest were bicyclists. More than half (55%) of the casualties befell on urban highways, while 28% occurred on highways outside the main cities. The vehicle-person mechanism of crash represents the highest percent (37%) followed by vehicle-object (33%). Vehicle-vehicle crashes were manifested in 30%.

**Transportation of injured people and the hospital arrival delay**: About 71% of casualties were transported to hospitals by a taxi and only 13% of casualties were transported by ambulance. The majority (83%) of casualties had been presented to the hospital in the same day of the accident; 42% of casualties presented within the same hour of the accident. The delay in hours ranged from 0 to 154 hours with a mean (SD) of 10 (27) hour.

**Risk factors**: Haddon's matrix and WHO injury surveillance guideline was used to classify the risk factors:

**During accident activity**: Injured people were asked of their activities at the time of the accident. About 60% of the patients were in vehicles whether drivers or occupants (31% on motorcycles and 29% in cars).

**Behavioral risk factors**: None of the victims wore the seat belt in case of 4-wheeled vehicles' users nor the helmet in case of 2-wheeled vehicles' users. Poor driving skills were involved in 133 (85%) casualties. Of the 133 cases, the most risky behavior involved was furious driving (44%), followed by the reverse directional driving (33%) and careless driving (23%). Occupants and pedestrians risky behavior were involved in 19% of the casualties collected. Of those casualties, careless pedestrians occurred in 70% of cases and clinging passenger in 30% of cases

**Vehicles' risk factors**: The total number of the vehicles involved in accidents documented is 148 vehicles. However, factors related to the vehicles contributed to 17 (12 %) RTCs. Broken brakes represented the highest percent (71%) followed by tire explosion (24%) and flat tires (5%).

**Environmental risk factors**: The environmental factors contributed to 47 (36 %) casualties. Among the environmental factors addressed, slippery roads of a non-weather causes represented 40% of the factors followed by the turns without road signs (30%). Potholes on the roads contributed to 15% of the casualties presented.

**Casualties' clinical data**: The documented accidents during the study period were resulted in a total of 266 associated casualties. Of these casualties, 156 were captured by the study and 14 died on roads. Table 2 shows the distribution of 156 casualties by the severity of the outcome and the Emergency Department (ED) management outcomes. Of the 156 casualties, 17% had severe injuries and needed ICU admission. Regarding the outcome of the Emergency Department (ED) management, one person died on arrival and 61 (39%) needed admission. Moderate and severe injuries that needed admission (61 cases) were followed until 48 hours post the accident. After 48 hours of the accident, 39% of patients ended with disability due to fractured limbs, 30% were not improving and their conditions were deteriorating, 18% had recovered and 5% died.

**Table 2:** The distribution of 156 casualties according to the severity of the outcome and the Emergency Department (ED) management outcomes

Variable	n	%
**The severity of injury**		
Minor or superficial (e.g. bruises, minor cuts)	93	59.6
Moderate requiring some skilled treatment (e.g. fractures, sutures)	35	22.4
Severe requiring intensive medical/surgical management	26	16.7
No apparent injury	2	1.3
**Management at the Emergency Department**		
Treated and discharged	92	59.0
Admitted	50	32.1
Referred to another hospital	11	7.1
Referred for out-patient follow up	2	1.3
Died	1	0.6
**Outcomes for admitted cases after 48 hours of the accident (61 cases)**		
Disability	24	39.3
Unstable and deteriorating	18	29.5
Complete recovery	11	18.0
In intensive care unit but stable	5	8.2
Death	3	4.9

**Figure 1:**
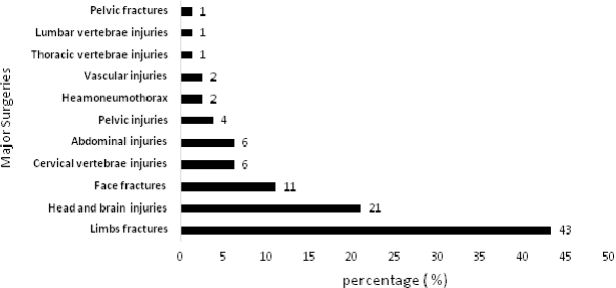
The distribution of the 61 admitted cases according to the 81 executed major surgeries

**Figure 2:**
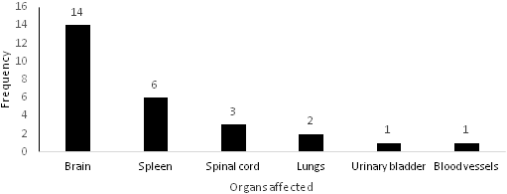
The distribution of the internal organs affected by road traffic crashes (n = 27)

**Executed major surgeries and types of injury**: For those who needed admission due to severe and moderate injuries (61 cases), 81 major surgeries in different parts of the body have been performed (Figure 1). Lower limbs represented 31% of the total injured body parts followed by head (29%) and upper limbs (20%). The most common injury were bruises and superficial wounds (74%), followed by fractures in 32% of the cases and internal organs injuries in 16% of them. Fractures occurred in 50 cases. However, the total number of fractures resulted from the accidents was 57 fractured bones as some patients had more than one fractured bone. Lower limbs fractures represented the main injury in 2-wheeled users. The number of casualties with internal organs injuries were 25. However, the total organs affected was 27 as some patients had multi-organs injuries. Figure 2 shows that the most affected internal organ was the brain (52%) followed by the spleen (22%).

## Discussion

The study captured 156 casualties from 128 accidents. The total number of the casualties affected by those accidents was 266. Of those, 156 presented to the study site. The total number deaths from the mentioned accidents was 18. The majority (78%) of deaths occurred at the site of the accidents. A similar finding was reported in Kenya study [6]. Since most deaths occurred pre-hospital, many might be preventable if they got the proper first-aid services. The mean age of victims in our study was 23 years, which is less than that noticed in other studies where the mean age was more than 30 [7-9].This study revealed that the highest number of casualties were 20-29 years old (34%), a finding that is consistent with the results of other studies in India and Nepal [7, 8, 10]. The next most affected age groups were the younger age groups (10-19 years old (20%) and less than 10 years old (19%). This finding was not seen in other studies that found fewer casualties in the extremes of age [7-14]. However, all the mentioned studies agreed with our study on that people less than 40 years old were at high risk of being injured. This finding reflects that people in productive age were mostly affected by RTIs, which adds a serious economic loss to the community. The high incidence in children raises the issue of parental care to their kids. The current study showed those males were more likely to be affected than females (male-female ration of 4.6:1). The same ratio was reported by hospital-based studies from Western Maharashtra, India [7, 15]. Although different ratios were reported by other studies, all agreed that male are more likely to be affected than females by more than three times [8, 16-19], probably due to more exposure and their risk taking behaviors.

However, the outcomes were more severe in females due to many factors; most probable one is that the majority of severe cases were among 4-wheeled passengers and those were tending to be females most often. This study showed that the working groups; employed (24%), laborers and farmers (19%) and business owners (5%) represent the most affected populations. Many other studies stated that laborers are the most affected working groups while other studies stated that business owners have higher risk (201). Risky driving behaviors contributed to 85% of the casualties. Furious driving represented 37% of these behaviors. A recent study from UAE ranked speed as the first unsafe behavior among drivers13. For pedestrians and passengers, their risky behaviors contributed to 19% of the casualties. The most risky behavior reported was pedestrians' carelessness. Head injuries were more common among the drivers and the passengers of 4-wheeled cars, while lower limbs injuries were more common among motorcycles riders and passengers. The same pattern was reported in many studies from different countries [8, 10, 19, 20]. It is evident that traffic-related trauma exerts a considerable burden on the already constrained health care resources in developing countries [6]. It was estimated by WHO that 100% of severe, 50% of moderate and10% of mildly injured persons need long-term rehabilitation services. A few hospital-based studies revealed that disabilities persist for a long time among 20%-40% of people discharged after an RTI [6, 15]. Consistent with this, our study showed that 39% of patients with moderate or severe injuries needed ended with disability. Several human and environmental risk factors had contributed to RTIs. Not using seat belt among the 4-wheeled users and helmet among the 2-wheeled users is one behavior that appeared to be of interest in terms of prevention of injuries. This critical finding should elicit the urgent need for road safety laws' enforcement in the country regarding the safety measures. Another important finding was that none of the victims had received any first aid services at the site of the crashes. This raises the issue of the preparedness of the health system especially that most deaths occurred pre-hospital and those might be preventable if victims got prompt and proper first-aid services.

## Conclusion

In conclusion, several personal, behavioral, environmental, and vehicles related factors had contributed to RTIs in Yemen. The burden of RTIs in Yemen in terms of disability and mortality is high. A critical first step toward improving road safety conditions is establishment of a multi-sectorial injury surveillance system. In addition, there is a need to improve access to adequate pre-hospital and hospital trauma care for road crash victims. Building the capacity for essential trauma care at public health facilities would benefit those vulnerable population groups

### What is known about this topic

- Road traffic injuries (RTIs) are the eighth leading cause of death globally and the leading cause of death for young people aged 15-29;
- The road traffic death rate in Yemen in 2013 was 23.7 deaths per 100,000 population;
- Research on road traffic injuries in Yemen is scarce and limited.

### What this study adds

- The majority of road traffic injuries in Yemen affected people less than 30 years old;
- Several personal, behavioral, environmental, and vehicles related factors had contributed to RTIs in Yemen.
